# Cigarette smoke and electronic cigarettes differentially activate bronchial epithelial cells

**DOI:** 10.1186/s12931-020-1317-2

**Published:** 2020-03-12

**Authors:** Christian Herr, Konstantinos Tsitouras, Julia Niederstraßer, Christina Backes, Christoph Beisswenger, Li Dong, Loïc Guillot, Andreas Keller, Robert Bals

**Affiliations:** 1 Department of Internal Medicine V – Pulmonology, Allergology and Critical Care Medicine, Saarland University, D-66421 Homburg, Germany; 2 Clinical Bioinformatics, Saarland University, University Hospital, 66123 Saarbrücken, Germany; 3 Department of Clinical Laboratory, Shanghai Tongji Hospital, Tongji University School of Medicine, Shanghai, 200065 China; 4 Sorbonne Universités, UPMC Univ Paris 06, INSERM, Centre de Recherche Saint-Antoine (CRSA), 75012 Paris, France

## Abstract

**Background:**

The use of electronic cigarettes (ECIGs) is increasing, but the impact of ECIG-vapor on cellular processes like inflammation or host defense are less understood. The aim of the present study was to compare the acute effects of traditional cigarettes (TCIGs) and ECIG-exposure on host defense, inflammation, and cellular activation of cell lines and primary differentiated human airway epithelial cells (pHBE).

**Methods:**

We exposed pHBEs and several cell lines to TCIG-smoke or ECIG-vapor. Epithelial host defense and barrier integrity were determined. The transcriptome of airway epithelial cells was compared by gene expression array analysis. Gene interaction networks were constructed and differential gene expression over all groups analyzed. The expression of several candidate genes was validated by qRT-PCR.

**Results:**

Bacterial killing, barrier integrity and the expression of antimicrobial peptides were not affected by ECIG-vapor compared to control samples. In contrast, TCIGs negatively affected host defense and reduced barrier integrity in a significant way. Furthermore ECIG-exposure significantly induced IL-8 secretion from Calu-3 cells but had no effect on NCI-H292 or primary cells. The gene expression based on array analysis distinguished TCIG-exposed cells from ECIG and room air-exposed samples.

**Conclusion:**

The transcriptome patterns of host defense and inflammatory genes are significantly distinct between ECIG-exposed and TCIG-treated cells. The overall effects of ECIGs on epithelial cells are less in comparison to TCIG, and ECIG-vapor does not affect host defense. Nevertheless, although acute exposure to ECIG-vapor induces inflammation, and the expression of S100 proteins, long term in vivo data is needed to evaluate the chronic effects of ECIG use.

## Background

The contribution of exposure to cigarette smoke (CS) to the development and progression of chronic obstructive pulmonary disease (COPD), cancer, and cardiovascular diseases is widely recognized [1, 2]. Electronic cigarettes (ECIGs) are commercially available since 2004, but patents for similar devices reach back to 1965 [3, 4]. ECIGs are devices that produce a vapor by heating a liquid [3] that usually contains a mixture of glycerol, propylene-glycol, water, flavors, and different concentrations of nicotine. The flavors cover a broad range of tastes from fruit or spices, to different brands of tobacco. An intense scientific and political discussion about the toxicity and potential harm reduction of ECIGs is ongoing.

Glycerol-propylene-glycol-water mixtures have been in use for a long time as artificial fog in aviation emergency training and entertainment business, but only a few studies about possible side effects exist before the emergence of ECIGs [5, 6]. Several studies have analyzed the effects of ECIGs on lung cells with the goal to evaluate toxic effects on cells and tissues [7]. A number of negative outcomes on different tissue culture systems (cell death, impaired repair, oxidative stress) have been reported [7–9]. In parallel, several studies compared ECIGs with TCIGs and often found decreased acute toxicity [10–16]. ECIG-vapor induced DNA-strand breaks in vitro [17] that are known to be induced by oxidative modifications of DNA by free radicals [18]. Several studies showed increased cytotoxicity and oxidative stress of flavored ECIG-vapor in vitro and in vivo [8, 19–21]. Additionally, it has been shown that ECIG-vapor may damage the pulmonary endothelial barrier and induce pulmonary neutrophilic inflammation [22, 23]. A recent study showed differences in gene expression in differentiated bronchial epithelial cells between TCIG- and ECIG-exposed cells, with and without nicotine [24], and showed differences in gene expression signatures in various pathways like phospholipid and fatty acid triacylglycerol, which were significantly enriched after ECIG-exposure.

In the present study we aimed to compare the acute effects of ECIG-vapor and TCIG-smoke on inflammation, host defense and cellular activation of human airway epithelial cells. We used an in vitro TCIG-exposure model [25] and adopted it to the vaporization of ECIG-liquid. Although we are aware that the composition of TCIG-smoke, besides nictotine and glycerol, is highly different from ECIG-vapor, we normalized the amount of ECIG-vapor to the content of nicotine from our established TCIG-exposure model. This has not been done in previously published studies and was important for direct comparison of the effects of ECIG-vapor and TCIG-smoke exposure. We chose nicotine consumption as a normalization factor to account for the needs of smokers to meet their demands for nicotine, when switching between TCIGs and ECIGs.

## Material and methods

### Cell culture

The human lung adenocarcinoma cell line Calu-3 (ATCC HTB-55) was cultured in DMEM/F12 (1:1) and the human bronchial epithelial cell line NCI-H292 (ATCC CRL-1848) was cultured in RPMI medium (Life Technologies, Darmstadt, Germany) both supplemented with 10% fetal bovine serum and 1% penicillin-streptomycin (Life Technologies, Darmstadt, Germany). Primary human bronchial epithelial cells (pHBE) were isolated from large airways from samples optained from macroscopically healthy areas of resected lung samples during surgery as described before [26]. The primary cells used within this study were from 3 different donors of Caucasian origin to account for intra-individual differences. The isolation and use of human specimen was approved by the ethics committee of the Landesaerztekammer des Saarlandes. The primary cells were grown in airway epithelial cell growth medium with growth supplement (Promocell, Heidelberg, Germany) and 1% penicillin-streptomycin (Life Technologies, Darmstadt, Germany), passaged once and freezed for later use. The experiments were repeated three times, all cells were tested in regular intervals and found to be mycoplasm-free.

### Air-liquid interface culture

The cells were seeded on 12-well transwell plates (Corning Inc., Kennebunk, ME, USA) at a density of 2–2.5 × 10^5^ cells / well. All experiments were performed on plates with a pore size of 0.4 μm except for barrier analysis, where membranes with 3 μm pore size were used [26]. After reaching confluence, the medium was changed to serum-free growth medium in the lower compartment and removed in the upper compartment. For primary cells after air-lift the medium was changed to antibiotic-free DMEM/F12 (1:1), supplemented with 2% Ultroser-G (Pall Life Science, Fribourg, Switzerland). Cell lines were used after reaching a transepithelial resistance of > 530 Ω/cm^2^ and primary cells after approaching more than 1000 Ω/cm^2^.

### Culture conditions of bacteria

*Pseudomonas aeruginosa* PAO1 (PAO1) was cultured on LB-agar plates (Roth, Karlsruhe, Germany) overnight. 5 mL LB-medium was inoculated with a single colony and cultured overnight (300 rpm, 37 °C). On the next day the 25 mL fresh medium was inoculated with bacteria from the overnight culture to yield an OD_600_ ≤ 0.3. The cells were cultured under agitation until they reached an OD_600_ of 1. For infection experiments with viable bacteria, PAO1 were diluted 1:10000 and applied in 15 μL to the apical surface of the air lifted cultures. For heat inactivation the undiluted solution was incubated for 5 min at 95 °C, stored in aliquots at − 20 °C and used for experiments in a dilution of 1:50 (approximately 53.4 × 10 ^6^ CFU/well).

### Cigarette smoke exposure

Differentiated air-liquid interface (ALI) cultures were exposed to volatile cigarette smoke form TCIGs as described earlier [25, 27]. Briefly, the ALI cultures were placed inside a modular incubator chamber (Billups-Rothenberg, Del Mar, CA, USA) that was placed inside a standard cell culture incubator. The incubator chamber was connected to a small membrane pump (Laboport N86 KN.18, KNF Neuberger, Freiburg, Germany) (for detailed information see supplementary data), that produced a negative pressure to conduct the smoke from commercially available Marlboro 100 s cigarettes to the ALI cultures placed inside the incubator box in the cell culture incubator. The negative pressure was adjusted to burn 3 cigarettes in 15 min uniformly. The smoke was mixed with humidified air from the incubator and conducted to the incubator chamber containing the ALI cultures.

### Electronic cigarettes

**Fig. 1:**
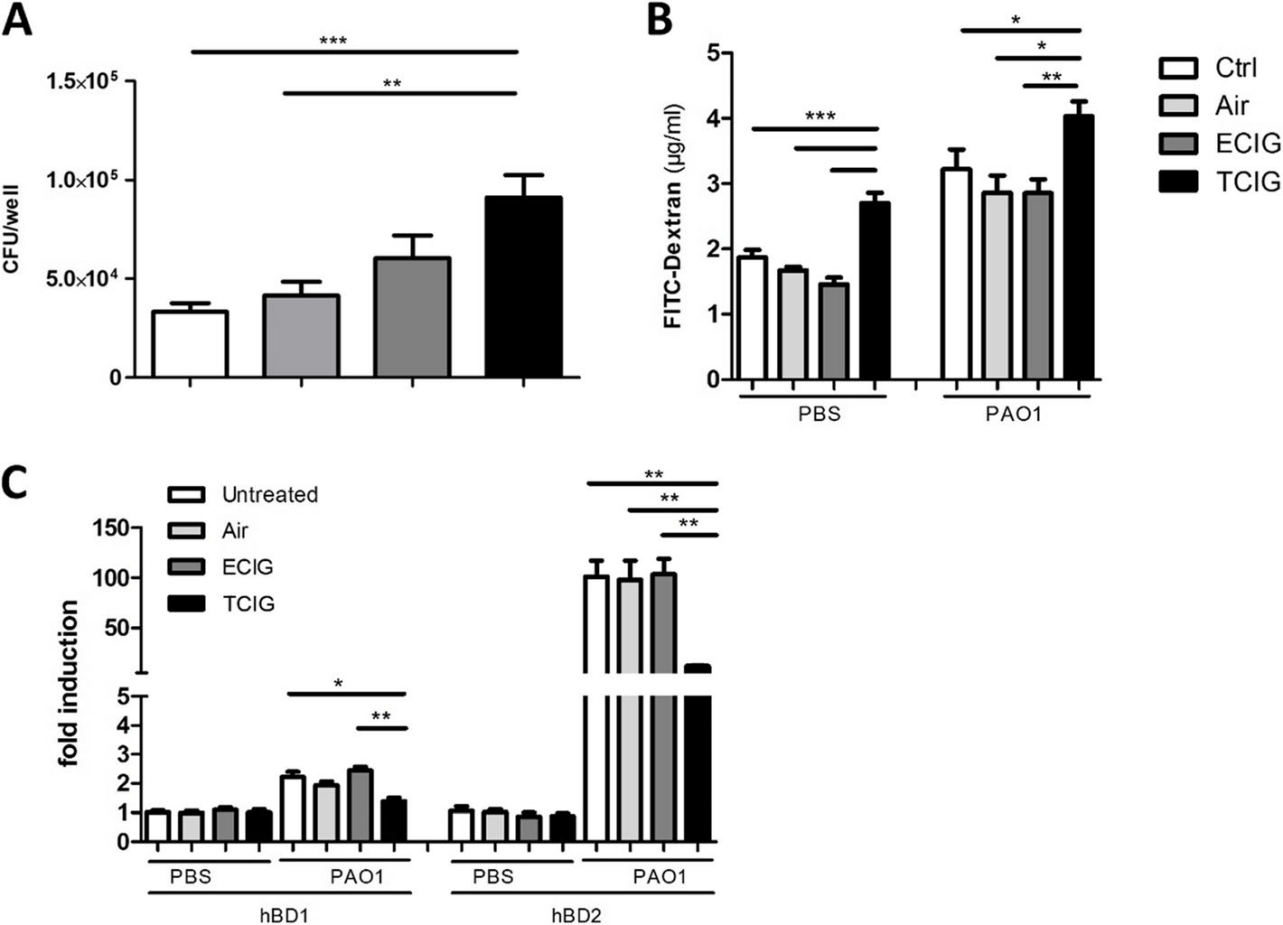
TCIG-exposure and ECIG differentially inhibit host defense. Calu-3 cells on transwell plates were exposed to TCIG or ECIG and infected with PAOI. 6 h after exposure and infection the highest number of bacteria was collected from TCIG-exposed cells, while ECIG-exposure only caused a moderate increase in bacterial counts **(a)**. The treatment with TCIG induced an increased translocation of FITC-Dextran from the apical to the basolateral compartment, which is independent of infection with PAOI **(b)**. The reduced host defense of TCIG-exposed samples correlated with a reduced expression of the antimicrobial peptides hBD1 and hBD2, which is induced after bacterial stimulation and not influenced by ECIG-vapor **(c)**. *N* = 6, one-way ANOVA, Tukey-Kramer adjusted post-hoc t-test, * *p* < 0,05, ** p < 0,01, *** p < 0,001

The ECIG-vapor was produced by a commercially available ECIG (steamo nova2, steamo GmbH, Leipzig, Germany). The ECIG consisted of a 3.5 mL refillable plexiglas tank and a 2.2 Ohms heating element that was operated at 2.6 Volts. The heating element was replaced after 10 uses. The cartridge was placed inside the cigarette holder of the custom-developed ECIG-device (Suppl.Fig. 1) and connected to a slightly modified exposure setup as described above. Briefly, a small ventilator was placed on the bottom of the incubator chamber to distribute the ECIG-vapor in the chamber. Commercially available liquid (60% propylene glycol, 30% glycerol, 10% water) without flavor and with 18 mg/mL nicotine was used (Dampfdorado, St. Ingbert, Germany). The ECIG-vapor was produced in intervals of 3 s each 29 s for a total duration of 15 min, which is similar to human “vaping” behavior [28, 29]. The concentration of ECIG-vapor was adjusted by regulating the airflow throught the ECIG to be equal to the amount of nicotine that is contained in 3 Marlboro 100 s cigarettes used for the TCIG-exposure. The concentration of ECIG-vapor was calculated as the difference in weight of the ECIG-cartridge before and after exposure. Given the known concentration of nicotine in the liquid the amount of nicotine was calculated and compared to the concentration of nicotine in the cigarettes.

### Normalization of ECIG-vapor and TCIG-smoke

We decided to normalize both exposure procedures on the content of nicotine since this is an ingredient common to cigarettes and ECIG with well-documented addiction promoting activity and toxicity [30–33]. In our established TCIG-exposure protocol we used 3 Marlboro 100 cigarettes, burned each in 5 min. Each cigarette from this brand contains 0.8 mg nicotine; a TCIG-exposure regimen would therefor contain approximately 2.4 mg nicotine. The mean density of the ECIG-fluid was calculated as a mean of 1.111 mg/mL (1.102 mg/mL without nicotine, 1.12 mg/mL with 18 mg/mL nicotine). Therefore 1 mg ECIG-fluid contains 1.63*10^− 2^ mg nicotine. Based on this calculation 147 mg of the ECIG-fluid would equal approximately 2.4 mg nicotine from 3 cigarettes. We adjusted the airflow to 1 LPM (liter per minute) from the incubator and the ECIG respectively. Using a vaping-puff length of 3 s every 29 s, we typically consumed 150–160 mg ECIG-fluid over 15 min and reached a the calculated amount of 2.4 mg nicotine per exposure.

### Determination of barrier integrity

To analyse the epithelial barrier integrity, the translocation of dextran from the apical to the basolateral side of the cell layer was determined as described before [34]. Directly after exposures, 200 μL of 10 mg/mL fluorescein labeled isothiocyanate–dextran (FITC-Dextran, 70 kDa, Sigma-Aldrich, Munich, Germany) in PBS or PBS containing heat inactivated *P. aeruginosa* PAO1 were applied directly on cells. 100 μL of the basolateral solution was collected after 24 hours and the fluorescence was measured. The fluorescence intensity was calculated against a standard curve of known concentrations of FITC-Dextran.

### Measurement of IL-8

We determined the IL-8 concentration in the basolateral solution of the air-liquid interface before and after exposure, by enzyme-linked immunosorbent assay (ELISA) according to the manufacturer’s instructions (R&D Systems, USA, Minneapolis, MN). We used a TECAN Ultra 384 ELISA reader and the software Magellan (Tecan, Mainz, Germany).

### RNA extraction and qRT-PCR

Total RNA of cells was isolated by Nucleospin RNA Kit according to manufacturer’s instructions (Machery Nagel, Düren, Germany) and used for whole-genome gene expression array analysis (Illumina Expression Bead Chip, Illumina Inc., San Diego, California) or validation of the array by qRT-PCR. cDNA was synthesized with the RevertAid First strand cDNA Synthesis Kit (Thermo Scientific, Schwerte, Germany) and oligo dT_18_-primers. The expression of the different genes was quantified by using the SensiMix™ SYBR® & Fluorescein Kit (Bioline, Luckenwalde, Germany) and the C1000 Touch™ Thermal Cycler (Bio-Rad, Munich, Germany). The specific primer sequences used in this work can be found in the sumpplemtary part (metabion international AG, Planegg, Germany). The expression was quantified by the ΔΔct-method [35].

### Transcriptome analysis

Primary human bronchial epithelial cells from 3 different donors were mixed and cultured as described above. After differentiation on transwell plates, the cells were exposed to TCIG and ECIG. Twenty-four hours after exposure RNA was isolated according to the manufacturer’s instructions (Nucleospin RNA Kit, Macherey Nagel, Düren, Germany). The RNA was used for expression analysis on an Illumina HumanHT-12 v4 Expression Bead Chip according to the manufacturer’s instructions. The analysis was performed by the Institute of Clinical Molecular Biology (Kiel University, University Hospital Schleswig Holstein, Germany).

The raw expression matrix was quantile normalized with the R package preprocess core. We identified the 100 genes having the highest variance over all samples and generated a heatmap with the heatmap.2 package from gplots. The heatmap also provides a dendrogram showing the clustering of the samples into groups (hierarchical clustering, Euclidean distance, complete linkage). Furthermore, we computed the differentially expressed genes in the groups using the quantile normalized data from the microarray and selected all genes with signals having a limma adjusted *p*-value *p* < 0.05 after 1-way ANOVA with post-hoc t-test. Data with an overall standard deviation > 0.5 and a limma adjusted *p*-value p < 0.05 was row-normalized, log2-transformed, and used to compile a heatmap of significantly differentially expressed genes (ratio of the mean expression values). Deregulated genes with a limma adjusted *p*-value < 0.05 and a difference in gene expression > 1.3 or < 0.6 fold were selected and compared for genes commonly deregulated between the groups using a Venn diagram [36].

Gene interaction networks were constructed based on log2 transformed normalized data with a limma adjusted p-value < 0.05 and an overall standard deviation > 1.5. The data was clustered (UPGMA, Euclidian distance) and interaction networks were drawn with the contextual network analysis software NetWalker [37].

### Statistical analysis

The data with normal distribution is displayed as mean and standard-deviation and data with skew distribution is expressed as median and interquartile range. The difference between groups (*n* ≥ 3) of normally distributed data was determined by parametric one-way analysis of variance (ANOVA) with a post-hoc Tukey-Kramer test or (*n* < 3) Student’s t test. The chi-square test was used to test group differences of non-normally distributed data or category data. The statistical analysis was perforemd using GraphPad Prism software (GraphPad Software Inc., V 5.02, La Jolla, CA, USA). Results were considered statistically significant for *p*-values less than 0.05. The statistical analysis for the transcriptome data is described above.

## Results

### ECIG has no effect on host defense

TCIG smoke is known to inhibit the host defense activities of epithelial cells and is a major risk factor for respiratory tract infections [27, 34]. To determine whether ECIGs have an effect on epithelial host defense, we exposed human Calu3 cells to TCIG or the corresponding amount of ECIG-vapor and infected the cells with 1 × 10^3^ CFU *P. aeruginosa*. The number of viable bacteria recovered from TCIG-exposed cells was significantly higher than the number of bacteria from untreated infected controls (Fig. 1 A). Samples that were exposed to ECIG-vapor did not contain considerably more viable bacteria than the infected control samples.

The epithelial barrier is an essential structural component of innate immunity. The translocation of high molecular weight dextran from the apical to the basolateral transwell compartment indicates a leaky epithelial barrier. Twenty-four hours after infection with *P. aeruginosa,* a higher concentration of FITC-dextran was detected in the basolateral compartment as compared to the non-infected groups (Fig. 1 b). The highest concentration of FITC-dextran in samples comparing the infected and non-infected groups was measured in the TCIG-exposed samples. The treatment with ECIG-vapor did not increase the translocation of FITC-dextran into the lower transwell compartment (Fig. 1 b).

Another important component of the innate immune system consists of antimicrobial peptides like defensins. The stimulation with *P. aeruginosa* led to a moderate induction of human beta-defensin-1 (hBD1), while the expression of human beta-defensin-2 (hBD2) was highly upregulated (Fig. 1 C). The treatment with ECIG-vapor did not change the expression of hBD1 or hBD2. In contrast TCIG-exposure significantly inhibited the expression of hBD1 and hBD2 in the infected groups (Fig. 1 c), which correlated with a higher bacterial load recovered from TCIG-exposed cells (Fig. 1 a).

### ECIG-exposure induces inflammation in vitro

**Fig. 2:**
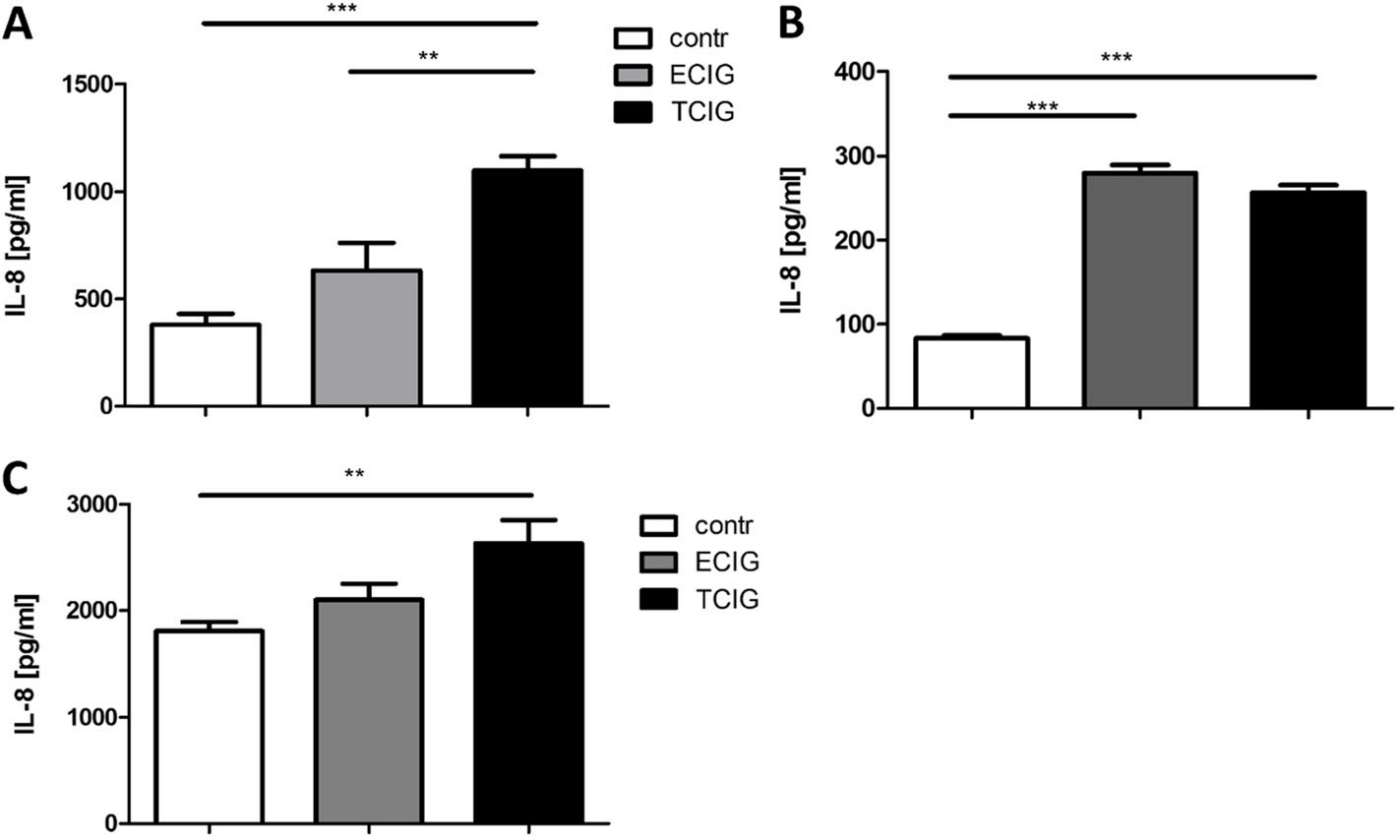
TCIG-exposure leads to a significantly increased synthesis of IL-8 in NCI-H292 cells **(a)**, Calu-3 **(b)**, and pHBE’s **(c)**. The treatment with ECIG-vapor induced a slight increase in IL-8 production in NCI-H292 **(a)** and pHBE **(c)**, but resulted in significantly increased IL-8 concentrations in Calu-3 cells **(b)**. N = 6, one-way ANOVA, Tukey-Kramer adjusted post-hoc t-test, * p < 0,05, ** p < 0,01, *** p < 0,001

TCIG-exposure of epithelial cells results in the induction of inflammation. Also, in the present study, TCIG-exposure leads to increased secretion of IL-8 from human bronchial epithelial cells (Fig. 2). In the NCI-H292 cell-line, the concentration of IL-8 after TCIG-exposure was significantly higher than in the samples treated with ECIG-vapor or the untreated controls (Fig. 2 a). In Calu-3 cells grown in the air-liquid interface we observed a significant increase of IL-8 secretion 24 hours after ECIG-exposure, which was similar to the amount found after TCIG-exposure (Fig. 2 b). In differentiated human primary bronchial epithelial cells, ECIG-vapor increased the secretion of IL-8 only moderately, while the exposure with TCIG induced a significantly higher production of IL-8 as compared to the mock exposed controls (Fig. 2 c).

### The gene expression pattern is distinct between TCIG and ECIG-exposed cells

To further characterize the transcriptional response of airway epithelium in response to exposure with ECIG and TCIG, we performed an array based analysis of gene transcription. We mixed equal numbers of human primary bronchial epithelial cells from three different donors and differentiated them in the air-liquid interface culture system. Twenty-four hours after exposure to TCIG, ECIG-vapor or room air (controls) RNA was extracted and used for expression analysis.

**Fig. 3:**
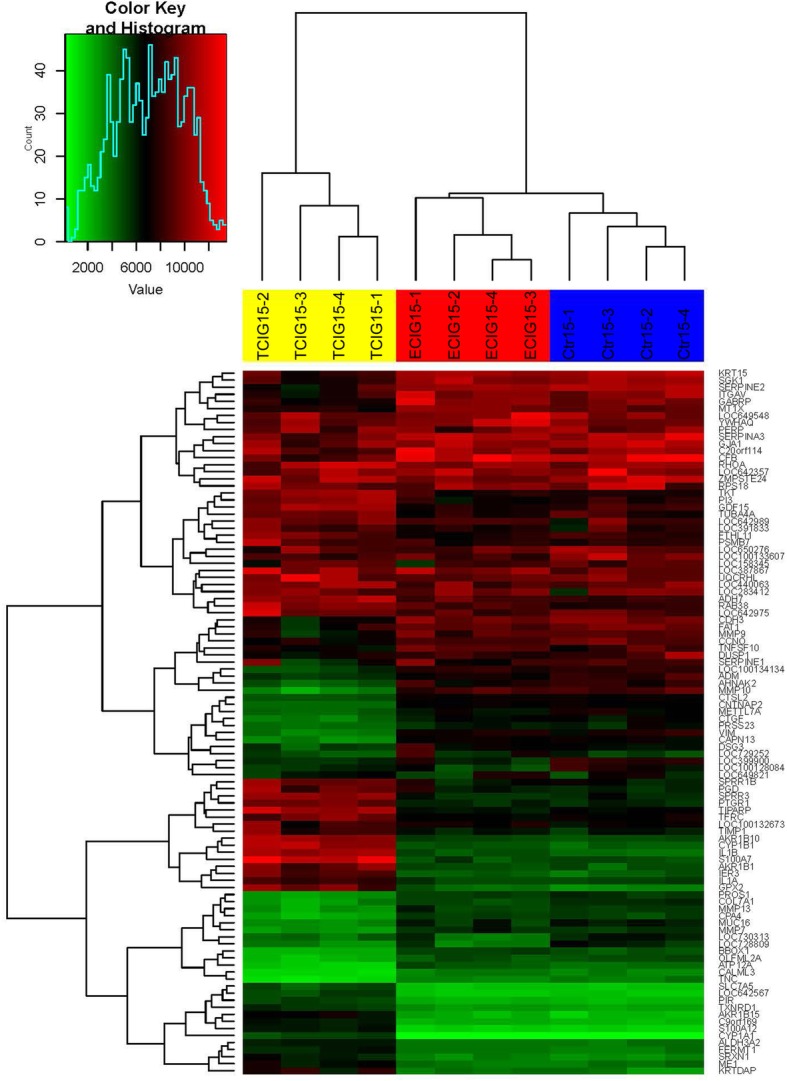
Heatmap of the normalized array expression data. We extracted the 100 genes with the highest variance in expression over all samples and performed a hierarchical clustering with complete linkage and Euclidean distance. The resulting shows clearly a separation of the three analyzed groups into distinct clusters. The expression of the ECIG group (ECIG15–1 - ECIG15–4) seems to be more similar to the control samples (Ctr15–1 – Ctr15–4), while the TCIG group (TCIG15–1 – TCIG15–4) shows blocks of genes that are clearly differentially expressed compared to the two other groups

First, we analyzed the normalized array data by clustering the samples using the 100 genes with the highest variance over all samples (Fig. 3). The heatmap clearly shows a distinction into two main clusters which separates the TCIG samples from the remaining samples. The second cluster is additionally divided into two clusters corresponding to the ECIG group and the control group. This clustering suggests that the TCIG samples have a very different expression pattern compared to ECIG and control samples, whereas the ECIG and controls can also be clearly separated but show a more similar expression pattern.

**Fig. 4:**
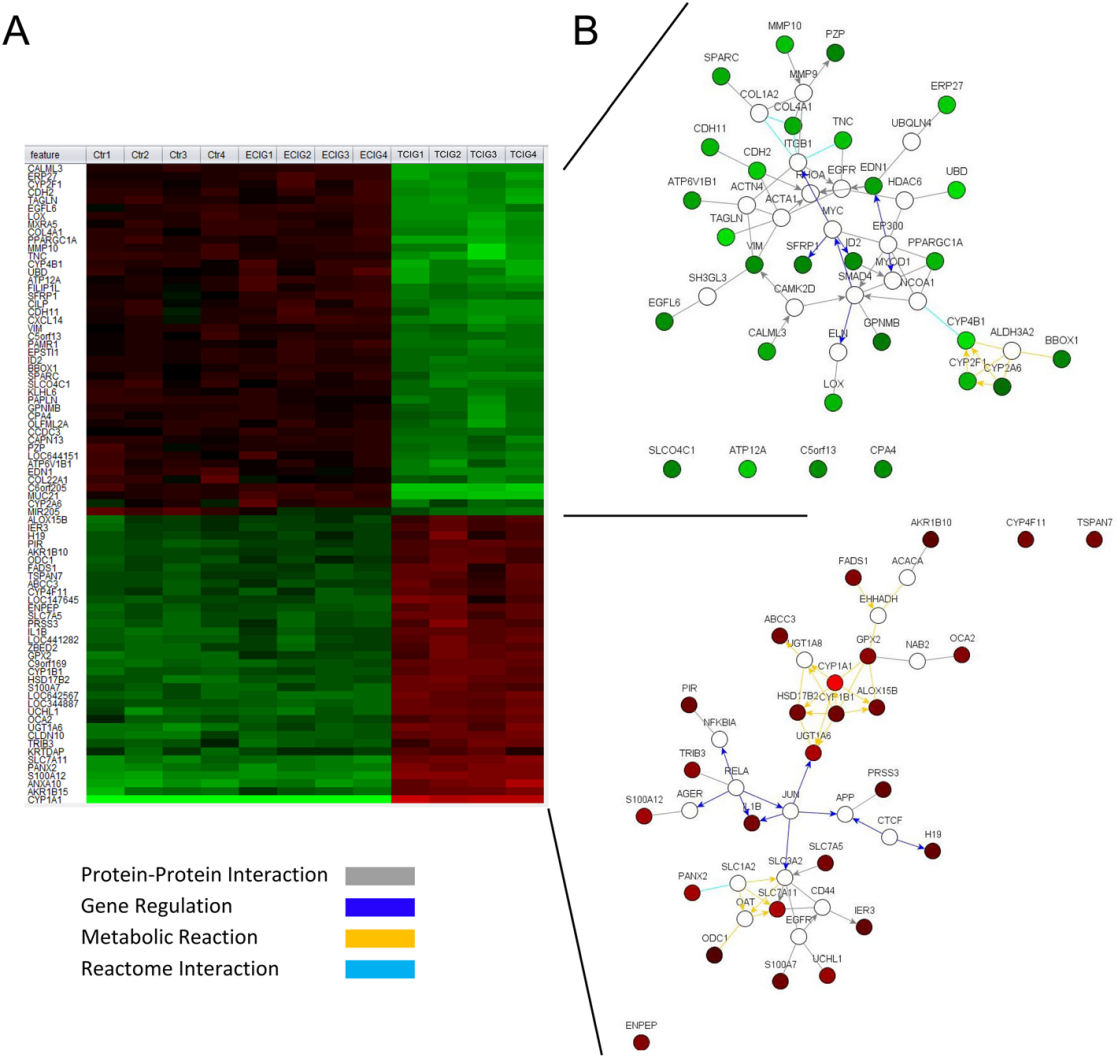
Heatmap from the significantly differentially expressed genes with a *p* < 0.05 (limma adjusted *p*-value) and a standard deviation > 0.5 over all samples (**a**). After clustering gene interaction networks were generated from the two resulting gene-expression clusters (**b**). Coloring of the nodes was done using the mean from TCIG-exposed samples (CS). The heatmap and the interaction networks were generated using Netwalker 1.0 [37, 38]. The lines connecting the nodes represent protein-protein interactions (grey), gene regulatory interactions (dark blue), metabolic reactions (yellow), and reactome interactions (light blue)

From the 31,422 transcripts analyzed, 2853 genes were significantly differently expressed (DEG) with an adjusted *p*-value < 0.05 (limma adjusted p-value) over all groups. Out of this group 80 genes showed a standard deviation greater 0.5 and were used for constructing the heatmap of significantly deregulated genes (Fig. 4 a) and the gene interaction network (Fig. 4 b). The heatmap clearly separates the samples into two clusters. One cluster contains ECIG-treated and control (Ctr) samples, while TCIG-exposed cells clearly separate from the other groups. Each cluster was used to construct gene interaction networks (Fig. 4 b) [38]. Each gene (node) is connected by edges (lines). Grey lines indicate protein-protein interactions, dark blue lines gene regulatory interactions, yellow metabolic interactions, and light blue reactome interactions. The data for this interactions were obtained by Netwalker from queries of HPRD (Human protein reference database), BIND (Biomolecular interaction database), MINT, BioGRID, IntAct, Reactome (obtained from Pathway Commons), NCI Pathway Interaction Database), KEGG, Human Metabolome Database (HMDB) and BiGG. Details about the mathematical model behind these networks are described by Komurov, et.al [37].

Among the genes upregulated by TCIG in the network, many show a REL-A and JUN dependent upregulation, which is known for pro-inflammatory stimulations like TCIG (Fig. 4 b). A number of genes were also upregulated that interact with cytochrome P450 CYP1A1, that is known to be involved in xenobiotic metabolism and the activation of aromatic hydrocarbons to carcinogens [39]. CYP1A1 showed the highest induction by TCIG compared to ECIG or Ctr-samples (28.1 fold, 31.5 fold, respectively, *p* = 7.42*10^− 7^).

**Fig. 5:**
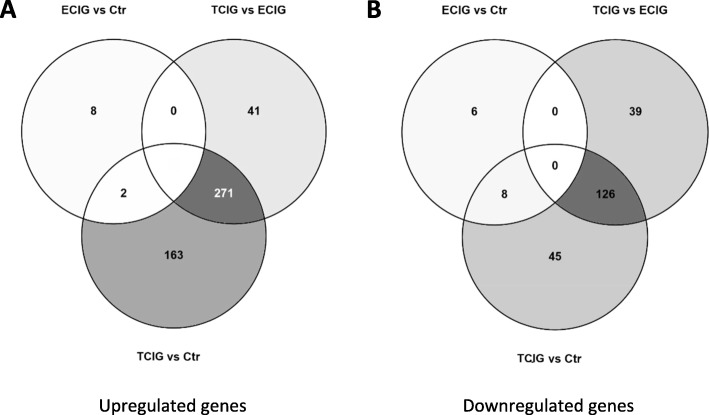
Venn diagram showing the number of genes shared and exclusively upregtulated **(a)** and downregulated **(b)** comparing ECIG/Ctr, TCIG/ECIG, and TCIG/Ctr. Genes with an adjusted p < 0.05 and an induction > 1.3 (**a**) or < 0.6 (**b**) were included

Among the DEGs with a limma-adjsuted *p*-value *p* < 0.05, the highest uniquely upregulated gene between ECIG and Ctr was CYP2A6 with a 1.37 fold increased expression (Table 1). To detect small changes, we decided to use a cutoff for analysis of 1.3 fold increase, although we are aware that this may not reflect a biologically relevant influence in acute exposure. Although significant, the most downregulated gene by ECIG compared to Ctr was an anti-sense ncRNA (ITPK1-AS1, C14orf85) with a 0.56 fold induction (Table 1). The highest significant repression was observed in the TCIG exposed samples compared to Ctr (MUC21) with a 0.29 fold increased expression (Table 1). In order to include the ECIG-exposed samples in the analysis, the cutoff for further analysis of downregulated genes was set to 0.6 fold downregulation, although these changes may be relevant in a chronic model. The compilation of induced and repressed genes into a Venn diagram [36] clearly shows that most genes are deregulated by TCIG smoke compared to ECIG and Ctr (Fig. 5). In contrast no gene is significantly deregulated by ECIG vs Ctr and ECIG vs TCIG. Only two genes, which are pseudogenes, are significantly upregulated by ECIG vs Ctr and TCIG vs Ctr (Fig. 5 a). A more detailed list of uniquely and commonly DEG is shown in the supplemental data (Supplementary Table 1).

**Table 1:** Differentially expressed genes

Exclusively upregulated genes	Symbol
Upregulated exclusively by ECIG compared to Ctr > 1.30 (range depicted 1.37–1.31, descending order)	CYP2A6, ZNF286A, APOBEC3B, WDR57, LPCAT2, LYPD2, CYP2B7P1, SLCO2A1
Upregulated exclusively by TCIG compared to ECIG > 1.30 (range depicted 1.52–1.33, descending order)	ARHGEF16, GDF15, PRDM1, RINT1, NTHL1, CYB5R2, CYP4F12, TNFRSF10A, RGS19, EDC3, APN5, SLC35C1, OTUB2, B3GNT8, ULK3, KRT18P17, CDC37L1, MAFF, CYP4F22, EHBP1L1
Upregulated exclusively by TCIG compared to Ctr > 1.30 (range depicted 1.56–1.45, descending order)	MAOB, IGSF5, CEP55, SPRED2, CICE, FKBP4, NIT2, MTHFS, NAPG, GDPD3, FEZ1, IFRD2, FOSL1, SHISA2, MVD, WDR72, AMN1, CYBASC3, GATC, IL1F5
Exclusively downregulated genes	
Downregulated exclusively by ECIG compared to Ctr < 0.60 (range depicted 0.56–0.59, increasing order)	C14orf85, NBPF8, CYP3A5, SNORA61, FLJ36131
Downregulated exclusively by TCIG compared to ECIG < 0.60 (range depicted 0.45–0.58, increasing order)	CYP2A6, SLCO2A1, HSPB3, TPM1, LYPD2, CA9, ALOX5AP, RTDR1, APCDD1, CYP4X1, C13orf30, SCARA3, MGC39900, RSPH9, MYLK, NGB, PPM1E, DNAI1, PTPRZ1, TSPAN8
Downregulated exclusively by TCIG compared to Ctr < 0.60 (range depicted 0.49–0.58 increasing order)	RYR3, DNAH12L, ODZ3, S100A3, AHNAK2, MIR221, ENC1, SMA4, TNFAIP8L1, MIR21, FLJ44342, TTC18, MEX3B, RGMA, FLJ23834, MAFB, MMP13, DNHD2, C1orf63, DCBLD1
Commonly deregulated genes	
Upregulated commonly in TCIG/ECIG and TCIG/Ctr > 1.30 (range depicted 37.5–2.50, descending order)	CYP1A1, ANXA10, S100A12,PANX2, SLC7A11, CLDN10, UGT1A6, AKR1B15, C9orf169, GPX2, UCHL1, HSD17B2, IL1B, TRIB3, SLC7A5, ZBED2, CYP1B1, ENPEP, S100A7, PRSS3
Upregulated commonly in ECIG/Ctr and TCIG/Ctr > 1.30 (range depicted 1.37–1.31, descending order)	(LOC100128899, LOC391019)
Downregulated commonly in TCIG/ECIG and TCIG/Ctr < 0.60 (range depicted 0.29–0.42, increasing order)	C6orf205, MUC21, TNC, CYP4B1, PPARGC1A, EDN1, TAGLN, LOX, CYP2F1, CALML3, ERP27, MMP10, CDH2, MXRA5, EGFL6, COL4A1, ATP12A, CDH11, UBD, CILP
Downregulated commonly in TCIG/Ctr and ECIG/Ctr < 0.60 (range depicted 0.51–0.59, increasing order)	FAM175A, MIR205, CATSPER2, GABRE, MGC16121

Only the top-20 genes are shown (where possible). For a complete listing see supplemental data

### Increased expression of redox and inflammation associated proteins induced by TCIG and ECIG

Among the 100 genes with the significantly highest gene expression variance (Fig. 3) and significantly DEGs (Fig. 4 a) several belonged to inflammation associated, tissue remodeling or antioxidant pathways respectively. Glutathione peroxidase-2 (GPX2 gene, Gpx2) is one example of such a gene, whose expression is significantly deregulated in the group comparison (ANOVA post-hoc t-test *p*-value, FDR adjusted: 2.3*10^− 4^; Supplementary Table 2) and shows an almost 3x up-regulation in the TCIG samples compared to the controls and a 2.3x up-regulation compared to the ECIG samples. The difference in expression for this gene is only 1.3x for ECIG compared to the controls (see Supplementary Table 3).

**Fig. 6:**
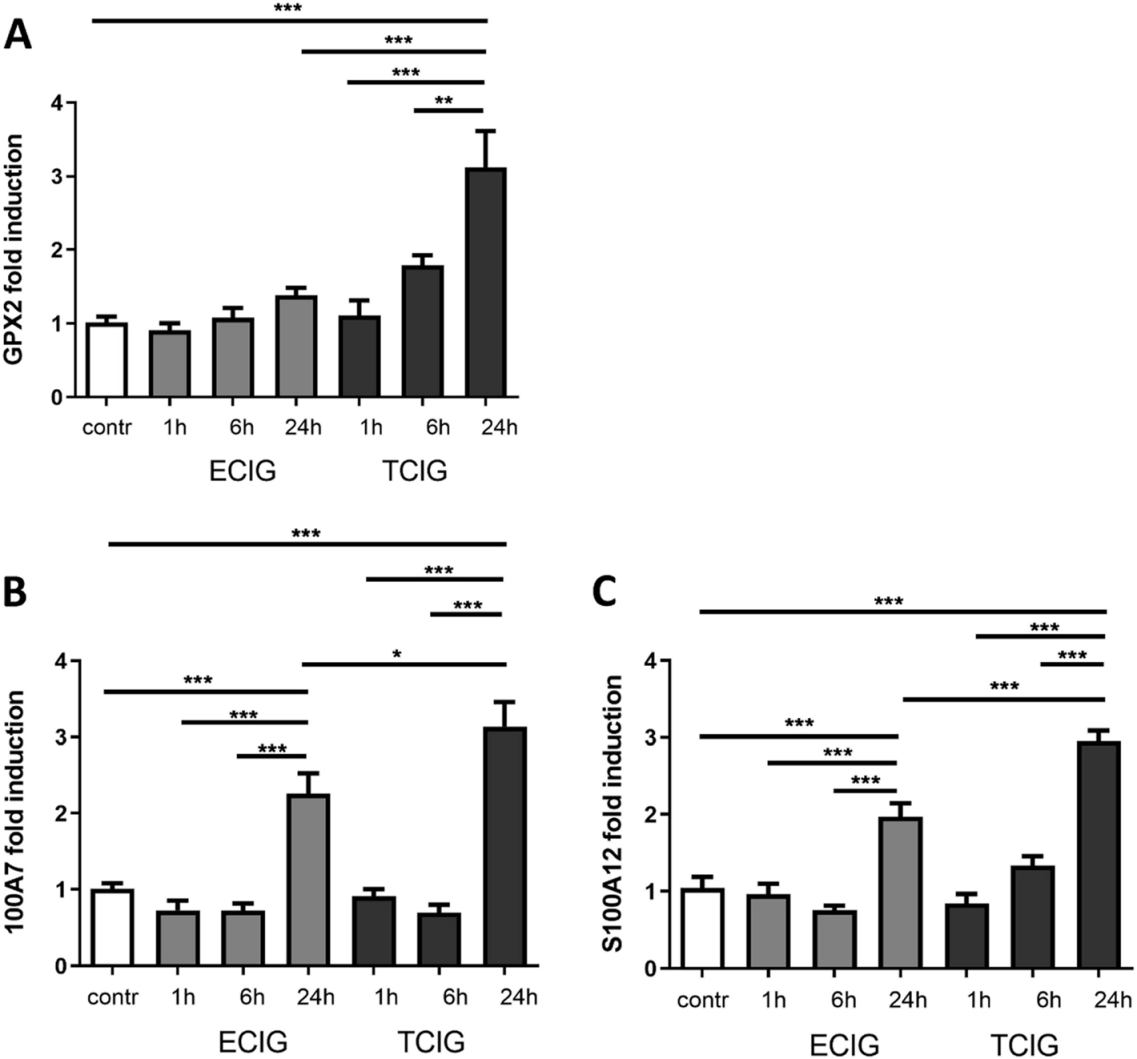
Validation of selected genes differentially regulated after array analysis by qRT-PCR in differentiated pHBE at different time points after exposure. The expression of GPX2 is only slightly upregulated by ECIG after 24 h but significantly induced in a time dependent manner after TCIG-exposure **(a)**. The expression of S100A7 **(b)** and S100A12 **(c)** is significantly induced 24 h after EZig-exposure and further increased significantly by TCIG at the same time-point. N = 6, one-way ANOVA, Tukey-Kramer adjusted post-hoc t-test, * p < 0,05, ** p < 0,01, *** p < 0,001

We confirmed the expression of GPX2 by qRT-PCR of differentiated pHBE that were TCIG- or ECIG-exposed after different time points (Fig. 6 a). While ECIG-exposure only induces a slightly increased transcription after 24 hours, the expression of GPX2 is significantly up-regulated 24 hours after TCIG-exposure (Fig. 6 a).

Based on the gene expression results from the array, S100A7 and S100A12 are among the 100 genes with the highest expression variance and are induced after TCIG-exposure.

S100 proteins comprise a family of calcium binding proteins involved in inflammation, host defense, and carcinogenesis [40].

Both genes are significantly deregulated (ANOVA, post-hoc t-test *p*-value FDR adjusted: 4.33*10^− 5^ and 2.03*10^− 7^; Supplementary Table 2) (adjusted p-value < 0.05) in the group comparison and upregulated in the gene interaction analysis. Although qRT-PCR shows a significant up-regulation of both S100 proteins 24 h after ECIG-exposure (Fig. 6 b-c), the gene-expression of S100A7 (Fig. 6 B) and S100A12 (Fig. 6 C) is further significantly increased 24 hours after TCIG-exposure.

The differences observed for ECIG-exposure after 24 hours may be due to differences in normalization procedures (different housekeeping genes) or hybridization efficiencies in the qRT-PCR compared to the Illumina bead array.

## Conclusion

The main finding of the present study is that ECIGs impact on the biology of airway epithelial cells with the release of inflammatory mediators but no overt reduction of antibacterial host defense. The acute toxic effect of ECIGs appeared to be less as compared to TCIG-exposure. Based on gene expression analysis, the expression patterns of TCIG-exposed cells were more different from sham exposed cells than ECIG-exposed cells.

Antimicrobial host defense is a fundamental function of epithelial tissues and comprises an active antimicrobial activity mediated by soluble molecules [41] and the formation of barriers that separate the inside from the outside. Exposure to TCIG is known to cause a breach of epithelial host defense [27] and impair the barrier function [42]. In the present study we did not observe a change in antimicrobial activity or barrier integrity after exposure of differentiated airway epithelium to ECIG-vapor. The results from the TCIG-exposed cells are in line with previous reports that TCIG-smoke leads to a defect in host defense and disruption of the epithelial barrier in vivo and in vitro [27, 34, 43]. We showed earlier that smoking is associated with reduced concentrations of hBD2 in airway secretions of patients with community acquired pneumonia, and that exposure of differentiated pHBE to volatile TCIG-smoke leads to a decreased expression and synthesis of hBD2 after infection with bacteria and an increased inflammatory reaction [27]. A study by Pace et al. showed that the expression of hBD2 is reduced in central airways of smokers and that the expression hBD2 positively correlates with lung function in COPD [44]. Exposure of epithelial cell lines with ECIG-vapor caused a decrease of antibacterial host defense and increased biofilm formation in [18, 45]. In a clinical study, ECIG use was associated with decreased expression of immune-related genes [46]. The application of ECIG-fluid, not vapor, to primary epithelial cells resulted in increased inflammation and susceptibility to virus infection [47].

Exposure of the lung to inhaled smoke or other noxious components is often associated with inflammation, which is involved in host defense, systemic reaction, and repair. There is a large body of data that exposure of airway epithelial cells to TCIG-exposure results in the release of a complex mixture of inflammatory mediators [42, 48–50] albeit the compound being responsible has not been identified so far. In the present study, TCIG-smoke leads to an increased synthesis and expression of pro-onflammatory mediators. In contrast, ECIG-treatment induced a lower stimulation of IL-8 sysnthesis or transcription of other inflammatory markers like S100A7 and S100A12. However, Calu-3 cells produced significantly elevated concentrations of IL-8 after ECIG-treatment compared to NCI-H292 or pHBE. Our results are partly in line with other studies published so far. It has been shown that the exposure of different bronchial epithelial cell lines to ECIG-vapor induced the release of IL-8 (NCI-H292-cells) [8, 51], increased IL-1β release and reduced cell proliferation (A549 cells) [52], and impaired barrier function of BEAS-2B cells (flavoring chemicals) [51].

To get a detailed view on the differentially regulated genes after TCIG- and ECIG-exposure, we analyzed the gene expression of differentiated airway epithelium after exposure. The changes of the transcriptome of the ECIG-exposed cells was much less as compared to that of TCIG-exposed cells. Glutathione peroxidase-2 (GPX2) is one example of a gene, whose expression is significantly deregulated in the group comparison of the top 100 most differentially expressed genes after ECIG-exposure. GPX2 reduces H_2_O_2_ to H_2_O and O_2_^−^, thereby oxidizing glutathione, which is reduced by glutathione reductase, that reduces NADPH to NADP [53]. The expression of GPX2 is slightly increased by ECIG-vapor and significantly more after TCIG-treatment. In contrast to GPX2, the expression of the S100-proteins S100A7 and S100A12 was significantly upregulated 24 h after ECIG-exposure. S100 proteins comprise a family of calcium binding proteins involved in inflammation, host defense, and carcinogenesis [40, 54]. S100A7 and S100A12 belong to a group of danger-associated proteins [55], which bind to cell surface receptors like RAGE and induce inflammation [56]. Additionally, S100A12 has been shown to induce the secretion of MUC5AC from airway epithelial cells [57]. Among the genes with the highest variance in gene expressen (Fig. 3) and the significantly upgregualted genes (Fig. 4) in the TCIG-group we also found IL1-β, another prominent pro-inflammatory mediator. A recent study applied RNA-seq analysis of differentiated airway epithelial cells and found similar impact of ECIG-vapor: The effects were detectable but less as compared to conventional TCIG. Various pathways such as the phospholipid and fatty acid triacylglycerol metabolisms were significantly enriched after ECIG-exposure [24]. Another study investigated the micro RNA (miRNA) response and the exposure of differentiated airway epithelial cells with ECIG-vapor resulted in the upregulation of oxaidative stress genes [58]. ECIGs also modify the metabolome of epithelial cells and showing significant changes partially overlapping with the effect of TCIG [59].

Our results indicate that ECIGs impact on epithelial biology with an effect on inflammation and metabolism. As compared to the exposure with TCIG, there was less impact on host defense, inflammation and gene expression. Other studies have shown that ECIGs induce antioxidant defenses and oxidative DNA damage in primary epithelial cells [60–63]. We used two different cell lines and pHBEs to account for different reactivity to TCIG and ECIG vapor. While NCI-H292 cells retain their original mucoepidermoid characteristics with nearly diploid chromosome counts, Calu-3 cells are highly transformed adenocarcinoma cells with hypotriploid chromosome counts [64]. The finding that ECIG-vapor induced a significant release of IL-8 only from the tumor cell line Calu-3 that was comparable with TCIG-exposed cells indicates that the vapor of ECIGs may induce inflammation in certain lung tumors, while it may not be pro-inflammatory for non-transformed bronchial epithelial cells (Fig. 2). We may speculate that this finding implicates that ECIG vapor could be more pathologic for individuals with pre-existing, yet silent cancerous lesions, but yet have to investigate this in a more detailed study.

The present study and other studies have limitations. In the first instance, all in vitro experiments provide only data on short term outcomes and do not allow to make predictions about the long-term effects of ECIG use. Therefore, it is also not possible to draw conclusions about the long-term safety or harm reduction potential of ECIGs. This study focus on specific cell lines and primary cells, while other studies focus on diverse other cellular systems.

The availability of flavors as additives for ECIGs and different types of vaporizing devices is growing and not regulated, and is not addressed in the present study. It has already been shown that falvours, especially cinnamon containing liquids, induce toxicity in vitro and in vivo [8, 19]. The variability in taste and nicotine strength is one of the features of ECIGs that surely attracts many people but on the other hand makes standardisation in research more complicated. In addition, the various exposure systems vaporize at different temperatures. This will lead to a different taste but also different chemical decomposition of ingredients. The boiling point of most of the ingredients found in flavored liquids is below 300 °C [20]. It has been shown, that the amount of formaldehyde, acetaldehyde and formalin is proportional to the output voltage of the device [65]. The devices offered currently range from 1 to 75 W (Joytech eGo AIO ECO 6–8 W, iStick Pico Melo 4 D22 1–75 W), producing temperatures of up to more than 300 °C.

The aim of the present study was to compare the impact of ECIG-vapor and TCIG on airway-epithelial cell biology. Comparing two physically different agents and trying to normalize is not straight forward. ECIGs produce a fine mist, containing the vaporized ingredients of the ECIG-fluid and trace amounts of emissions from the heating element. TCIG-smoke in contrast is a mixture of combustion-degraded ingredients from tobacco containing small particles, gas, and small amounts of humidity. Nicotine is one of the few ingredients that is common to TCIG-smoke and ECIG-vapor that is absorbed, and, what is even more important, with known toxicity and addictive properties. Normalizing on nicotine consumption therefore seems reasonable, since the user will be in need for certain amounts of nicotine, which will make the overall uptake between TCIG and ECIG comparable [66].

Only a few studies directly compare TCIG and ECIG in airway epithelial cells. One of the publications that closely reflects our setup and normalization procedures [67] used several dilutions of ECIG and TCIG and determined the concentration of nicotine after the exposure in the chambers. Their data show a significantly different gene expression pattern between ECIG- and TCIG-exposed samples when comparing conditions with similar nicotine concentration. In addition, they also showed an upregulation of many genes after TCIG-exposure, that were similar to our experiments (i.e. IL1A, IL1B, GPX2, CYP1A1, CYP1B1, S100A12). Although a slightly different setup was used [68], another publication showed that in comparison to TCIGs the vapor of ECIGs induced only minor changes in gene expression, although their smoking protocol used with ECIGs was more intense than the corresponding TCIG-exposure. In their 6 × 8 min TCIG-exposure protocol similar genes were upregulated like in our protocol (i.e. IL1A, IL1B, GPX2, CYP1A1, CYP1B1, S100A12) [68]. Although different protocols for ECIG-exposure and different cells were used, both studies agree with our findings, that TCIG-exposure induces a transcriptomic profile, which is different from ECIG-exposed cells and that the majority of differentially expressed genes can be found in TCIG-exposed samples.

In conclusion, ECIG-vapor has an acute effect on the biology of AECs. ECIGs had no significant effects on the secretion of chemokines or antimicrobial peptides after bacterial stimulation but induced the expression of S100A7 and S100A12. While the effects of ECIGs on epithelial cells appears to be less toxic as compared to TCIGs, in vitro results do not permit to draw conclusions about the long-term safety.

## Supplementary information

**Additional file 1:** **Supplementary methods.**

**Additional file 2:** Deregulated genes - pairwise comparisons with varinace.

**Additional file 3:** Goupwise comparison of gene expression.

**Additional file 4:** Deregulated gened - pairwise comparison raw data.

## Data Availability

All data is available upon request.

## Abbreviations

ALI: Air-liquid interface
CDNA: Complementary DNA
CFU: Colony forming units
COPD: Chronic obstructive pulmonary disease
CS: Cigarette smoke
Ctr: Control
DEG: Differentially expressed gene
DMEM: Dulbecco’s minimal essential medium
DNA: Desoxyribonucleic acid
ECIG: Electronic cigarette
ELISA: Ezyme linked immunosorbent assay
FITC: Fluorescein isothiocyanate
GAPDH: Glyceraldehyde 3-phosphate dehydrogenase
Gpx2: Glutathione peroxidase 2
HBD1: Human beta-defensin-1
HBD2: Human beta-defensin-2
JUN: Transcription factor, part of AP-1 Transcription factor
LB-agar: Lysogeny broth agar
LPM: Liter per minute
NcRNA: Non-coding ribonucleic acid
PBS: Phosphate buffered salined
PHBE: Primary human bronchila epithelial cells
QRT-PCR: Semiquantiative realtime polymerase chain reaction
REL-A: V-rel avian reticuloendotheliosis viral oncogene homolog A, p65 subunit of Nf-kB
RNA: Ribonucleic acid
RPMI-1640: Roswell Park Memorial Insititute medium
S100A12: S100 calcium binding protein A12, Calgranulin C
S100A7: S100 calcium binding protein A7, Psoriasin
TCIG: Traditional cigarette
