# Supplementary material for: Cigarette smoke and electronic cigarettes differentially activate bronchial epithelial cells
Source: Respir Res. 2020 Mar 12;21:67. doi: 10.1186/s12931-020-1317-2 (PMC7068890; doi:10.1186/s12931-020-1317-2)
Supplement: Supplementary file 1 — Additional file 1: Supplementary methods. [file 12931_2020_1317_MOESM1_ESM.docx]

**Supplementary Data and Figures:**

**Supplementary Figure 1**

**A**

**B**


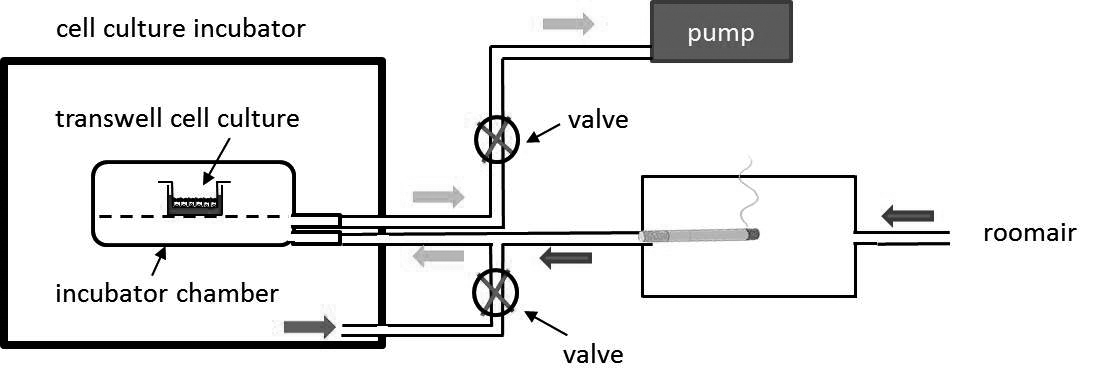

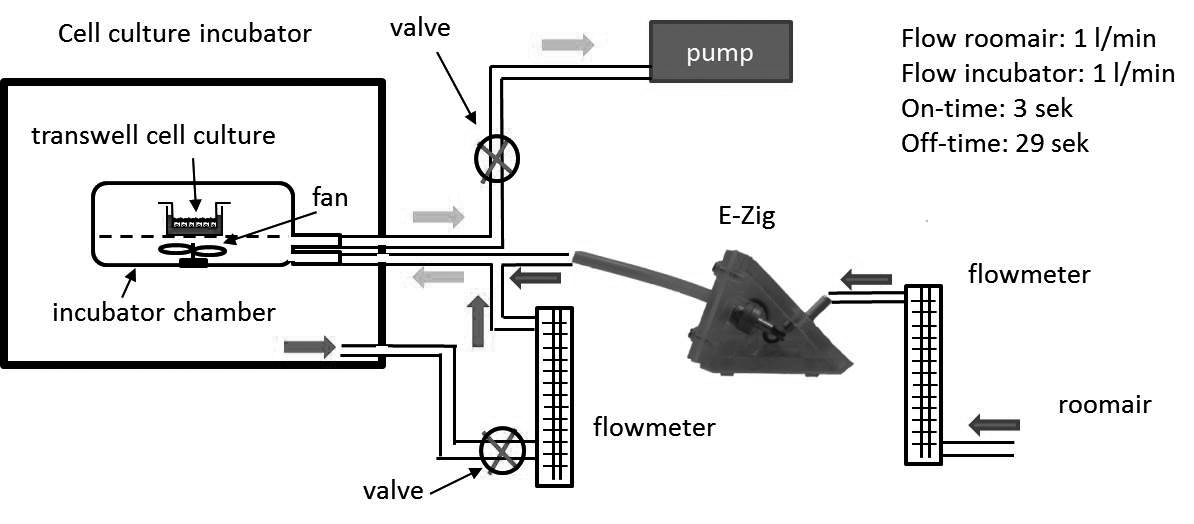

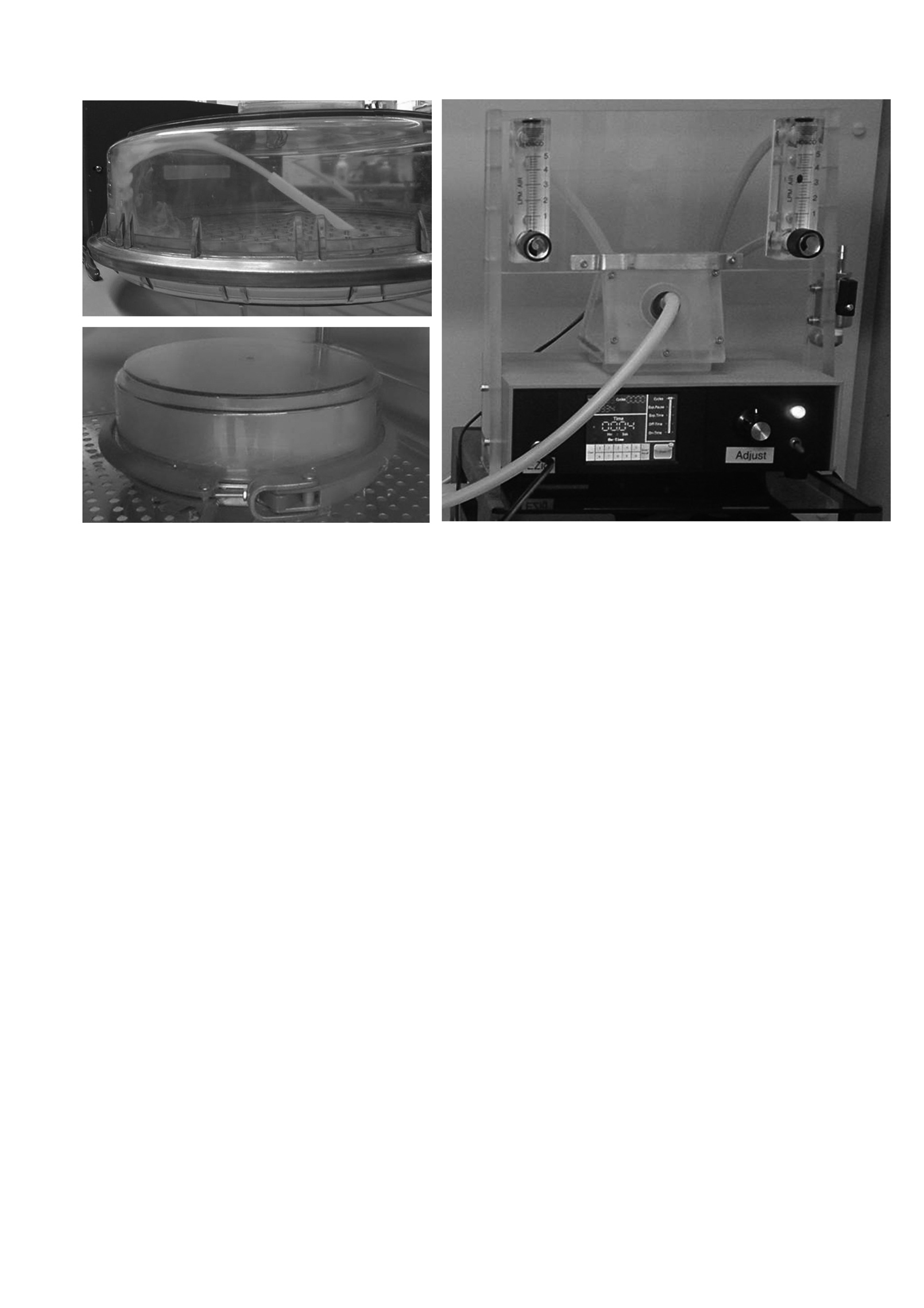


**CA**

**E**

**D**

**Supplementary Figure 1:** The *in vitro* exposure system. **(A)** The TCIG-exposure system as published earlier (10). A commercial cigarette is burned in a plexi glass box outside the cell culture incubator. Air from the incubator is mixed with TCIG-smoke and drawn over the cells, growing in conventional transwell plates in an incubator chamber inside the cell culture incubator. The flow can be adjusted to yield an equal mixture of TCIG and incubator atmosphere. The overall negative pressure is produced by a pump and can be regulated at a second valve. **(B)** Modified flow scheme for the use with ECIGs. Flow meters were added to control the flow more precisely. A small fan below the cell culture plate is needed to distribute the ECIG-vapor in the incubator chamber. **(C)** ECIG-vapor in the incubator chamber is quickly accumulated at the bottom of the chamber. **(D)** A fan was introduced in the chamber to distribute the vapor. **(E)** Microcontroller based ECIG-controller with flow meters and ECIG-cartridge. Total exposure time, on time, off time and number of cycles can be set on the touch screen. The voltage for the ECIG can be adjusted between 0-5 V.

**Supplementary methods:**

**Specific primers used in this work:**

| **hBD1** | **forward** | 5’-GCC TCA GGT GGT AAC TTT CTC A-3’ |
| --- | --- | --- |
|  | **reverse** | 5’-GCG TCA TTT CTT CTG GTC ACT-3’ |
| **hBD2** | **forward** | 5’-CAG CCA TGA GGG TCT TGT ATC T-3’ |
|  | **reverse** | 5’-GAA TCC GCA TCA GCC ACA GC-3’ |
| **Gpx2** | **forward** | 5’-ACT TCA CCC AGC TCA ACG AG-3’ |
|  | **reverse** | 5’-ATG CTC GTT CTG CCC ATT CA-3’ |
| **S100A7** | **forward** | 5’-CTT CCC AGC TCT GGC TTT TTG A-3’ |
|  | **reverse** | 5’-AGA CAT CGG CGA GGT AAT TTG T-3’ |
| **S100A12** | **forward** | 5’-TTC CTG TGC ATT GAG GGG TTA-3’ |
|  | **reverse** | 5’- AAT GCC CCT TCC GAA CTG AG-3‘ |
| **GAPDH** | **forward** | 5’-GGA AGG TGA AGG TCG GAG TC-3’ |
|  | **reverse** | 5’-GCA GTG ATG GCA TGG ACT G-3’ |

**Normalization of ECIG-vapor**

TCIG-smoke is a complex combustion mixture of tobacco and many other ingredients that had been included in cigarettes by the producers. ECIG-liquid mainly contains propylene-glycol, glycerol, and water. We decided to normalize both exposure procedures on the content of nicotine, since this is an ingredient common to cigarettes and ECIG with well documented addiction promoting activity (1-4). In our established TCIG-exposure protocol we used 3 Marlboro 100 cigarettes, burned each in 5 minutes. Each cigarette from this brand contains 0.8 mg nicotine, a TCIG-exposure regimen would therefor contain approximately 2.4 mg nicotine. The mean density of the ECIG-fluid was calculated 1.111 mg/ml (1.102 mg/ml without nicotine, 1.12 mg with 18 mg/ml nicotine). Therefore 1 ml ECIG-fluid contains 1.63*10^-2^ mg nicotine. Based on this calculation 147 mg of the ECIG-fluid would equal approximately 2.4 mg nicotine from 3 cigarettes. We adjusted the airflow to 1 LPM (liter per minute) from the incubator and the ECIG respectively. Using a puff length of 3 seconds every 29 seconds we typically consumed 150-160 mg ECIG-fluid over 15 minutes.

Supplementary Literature:

1. Arntzen, F. I. 1948. Some psychological aspects of nicotinism. *Am J Psychol* 61: 424.

2. Dani, J. A., and M. De Biasi. 2001. Cellular mechanisms of nicotine addiction. *Pharmacology Biochemistry and Behavior* 70: 439-446.

3. Chernyavsky, A. I., I. B. Shchepotin, V. Galitovkiy, and S. A. Grando. 2015. Mechanisms of tumor-promoting activities of nicotine in lung cancer: synergistic effects of cell membrane and mitochondrial nicotinic acetylcholine receptors. *BMC Cancer* 15: 152.

4. Spindel, E. R., and C. T. McEvoy. 2016. The Role of Nicotine in the Effects of Maternal Smoking during Pregnancy on Lung Development and Childhood Respiratory Disease. Implications for Dangers of E-Cigarettes. *Am J Respir Crit Care Med* 193: 486-494.
